# Pixel response‐based EPID dosimetry for patient specific QA

**DOI:** 10.1002/acm2.12007

**Published:** 2016-12-15

**Authors:** Bin Han, Aiping Ding, Minghui Lu, Lei Xing

**Affiliations:** ^1^ Radiation Oncology Department Stanford University Stanford CA USA; ^2^ Perkin Elmer Medical Imaging Santa Clara CA USA

**Keywords:** dosimetry, EPID, Monte Carlo, patient‐specific QA

## Abstract

Increasing use of high dose rate, flattening filter free (FFF), and/or small‐sized field beams presents a significant challenge to the medical physics community. In this work, we develop a strategy of using a high spatial resolution and high frame rate amorphous silicon flat panel electronic portal imaging device (EPID) for dosimetric measurements of these challenging cases, as well as for conventional external beam therapy. To convert a series of raw EPID‐measured radiation field images into water‐based dose distribution, a pixel‐to‐pixel dose–response function of the EPID specific to the linac is essential. The response function was obtained by using a Monte Carlo simulation of the photon transport in the EPID with a comprehensive calibration. After the raw image was converted into the primary incident photon fluence, the fluence was further convolved into a water‐based dose distribution of the dynamic field by using a pregenerated pencil‐beam kernel. The EPID‐based dosimetric measurement technique was validated using beams with and without flattening filter of all energies available in Varian TrueBeam STx™. Both regularly and irregularly shaped fields measured using a PTW 729 ion chamber array in plastic water phantom. The technique was also applied to measure the distribution for a total of 23 treatment plans of different energies to evaluate the accuracy of the proposed approach. The EPID measurements of square fields of 4 × 4 cm^2^ to 20 × 20 cm^2^, circular fields of 2–15 cm diameters, rectangular fields of various sizes, and irregular MLC fields were in accordance with measurements using a Farmer chamber and/or ion chamber array. The 2D absolute dose maps generated from EPID raw images agreed with ion chamber measurements to within 1.5% for all fields. For the 23 patient cases examined in this work, the average γ‐index passing rate were found to be 99.2 ± 0.6%, 97.4 ± 2.4%, and 72.6 ± 8.4%, respectively, for criterions of 3 mm/3%, 2 mm/2%, and 1 mm/1%. The high spatial resolution and high frame rate EPID provides an accurate and efficient dosimetric tool for QA of modern radiation therapy. Accurate absolute 2D dose maps can be generated from the system for an independent dosimetric verification of treatment delivery.

## 1 Introduction

The use of amorphous silicon (aSi) flat panel electronic portal imaging device (EPID) for online and offline dosimetric verification has been sought after over the years by several research groups and industrial companies.^1^, ^2^, ^3^, ^4^, ^5^, ^6^ For example, the Portal Dosimetry™ from Varian Medical Systems (Palo Alto, CA, USA) has been available for pretreatment QA.^1^ In this product, beams are directly applied to the portal imager and time‐integrated imaging data are acquired. By comparing the measurement with the calculation using the photon fluence from the treatment plan, a QA decision is made based on a series of criteria, such as the percentage difference, distance to agreement (DTA), and γ‐index analysis. Because the response of the portal imager is quite different from water, this approach is incapable of providing absolute dosimetric information. Instead, it only gives an indirect comparison of fluence. Mans et al.^2^ used the EPID to catch errors in routine clinical IMRT and 17 serious errors were detected among 4227 patients treated. McCurdy et al.^3^ investigated the dosimetric properties of an EPID operated in continuous acquisition mode for verification of dynamic and arc IMRT. Woodruff et al.^7^ and Liu et al.^8^ used the approach for pretreatment verification QA of VMAT. Asuni et al.^9^ and Lee et al.^10^ used EPID images to reconstruct in vivo 3D dose for Stereotactic Body Radiation Therapy (SBRT) QA. Recently, Nelms et al.^11^ and Bailey et al.^12^ investigated the use of EPIDose™ (Sun Nuclear Corporation, Melbourne FL, USA) for pretreatment QA. The EPIDose converts an EPID image to dose in water by convolving with an experimentally determined kernel to account for the difference in dose‐deposition kernels of the EPID and water. Because the detailed EPID response was not studied, for each MLC‐segmented field, an output correction factor must be calculated from MLC plan data and applied to the measurement, which may be a significant source of inaccuracy. Greer et al.^13^ developed an EPID‐based dose prediction model by incorporating MLC leaf effects for IMRT applications. The EPID dose kernel was calculated using an experimental method and is only specific to the Pinnacle treatment planning system. Warkentin et al.^14^ improved the approach with a convolution‐based calibration procedure, in which the physics response of the EPID was deduced from the combination of a Monte Carlo‐simulated dose deposition kernel in the EPID phosphor, and an empirically derived kernel describing optical photon spreading. Nicolini et al.^15^ had recently demonstrate the feasibility of using EPID dosimetry for flattening filter free (FFF) photon beams by means of the GLAaS methodology to validate it for pretreatment quality assurance of volumetric modulated arc therapy (VMAT), but EPID calibration data were obtained against ion chamber measurements. While all these studies indicated that the EPID is useful as a dosimetric tool, to the best of our knowledge, a complete and accurate method to convert MV photon beams physics response of the EPID to a water‐equivalent dose distribution has yet to be obtained with consideration of the generation and transport of the optical photons in the scintillators. Furthermore, there are little investigation adapting EPID for dosimetry of a high dose rate and small field radiation therapy (RT).

This work is thus devoted to develop a strategy of using a high spatial‐resolution and high frame rate a‐Si EPID for dosimetric verifications of various modalities of modern RT, including small FFF fields with high dose rate. In the next section, we introduce the setup of experimental data acquisition and the calibration of the system. The methods to deconvolve the primary fluence and water‐based dose are presented in Sec. 2B–C. Validation and application issues related to the implementation of the proposed method are discussed in Sec. 2D–E. We conclude in Sec. 4 with highlights of the study and future perspectives of EPID‐based dosimetric verification.

## 2 Materials and methods

### 2.A Overall system setup and data acquisition

A standalone portable XRD‐0822 AP20 a‐Si flat panel detector (PerkinElmer Inc, Sunnyvale, CA, USA) was used in this study. The size of detector was 20.48 × 20.48 cm^2^, with a matrix of 1024 × 1024 pixels, a minimum pixel size of 0.2 mm, and a maximum frame rate of 50 frames per second (fps). The images were acquired in “cine‐mode”, in which each individual frame was recorded over the entire beam‐on time and transferred through a Gigabit Ethernet network cable to a control computer for data analysis.

**Figure 1:**
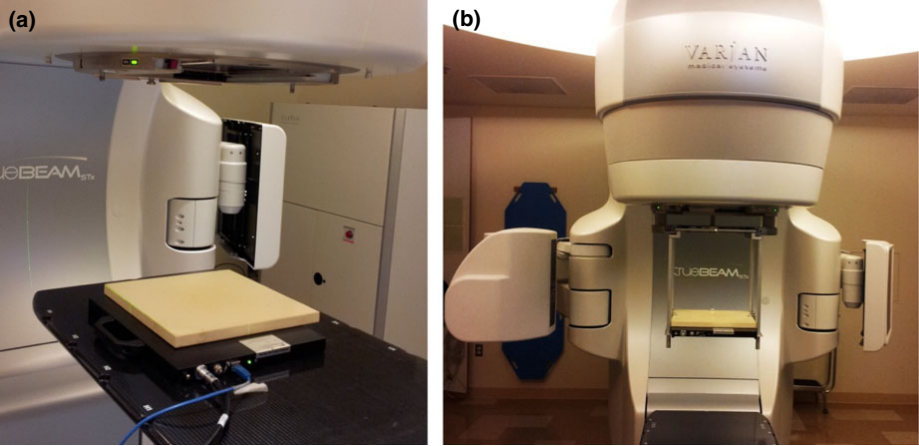
The (a) on‐couch stationary and (b) on‐head rotational settings for the EPID system.

Measurements were performed on a TrueBeam STx Linac (Varian Medical Systems, Palo Alto, CA, USA) for all five available photon energy modes: 6 MV, 10 MV, 15 MV beams with a flattening filter (WFF), and 6 MV and 10 MV flattening filter free (FFF) beams. Figure 1 shows the stationary and rotational settings for the EPID system. In a stationary setting (Fig. 1a), the EPID was placed on the treatment couch, useful for QA measurement of fixed gantry deliveries such as IMRT or any other type of dynamic treatment with the gantry angles of the contributing beams reset to 0°. In the latter case (Fig. 1b), a customized holder was used to mount the EPID on the linac head. The system is capable of measuring the dose at each gantry angle for a rotational arc delivery such as VMAT. For both settings, the EPID imager is placed beneath a 2‐cm thick PlasticWater^®^ (Computerized Imaging Reference System Inc, Norfolk, VA, USA) build‐up phantom for photon measurement. A source to detector distance (SDD) of 100 cm was used for both stationary and rotational settings.

Before image acquisition, a dark field (DF) image and a flood field (FF) image were acquired for offset and gain corrections. The offset correction took into account the dark current of each pixel and acquired with photon beam off. In order to create the offset correction image, an averaged image (${\bar{\text{EPID}}}_{\mathrm{DF}}$) of 300 frames of DF images had to be acquired and ${\bar{\text{EPID}}}_{\mathrm{DF}}$ would be subtracted from the incoming pixel data during acquisition time. To homogenize differences in pixel sensitivities, an FF gain correction was carried out at all available photon energies of 6 MV, 10 MV, 15 MV, and 6 MV FFF, 10 MV FFF beams by irradiating the EPID with the incident photon beam fully covering the entire detector‐sensitive field (20 ×20 cm^2^). To create the FF image, an averaged image (${\bar{\text{EPID}}}_{\mathrm{FF}}$) of 300 frames of offset‐corrected images has to be acquired. Each EPID‐measured raw image is corrected by using the following equation

(1) $${\text{EPID}}_{\mathrm{raw}}{|}_{\mathrm{corrected}}=\frac{{\text{EPID}}_{\mathrm{raw}}-{\bar{\text{EPID}}}_{\mathrm{DF}}}{{\bar{\text{EPID}}}_{\mathrm{FF}}-{\bar{\text{EPID}}}_{\mathrm{DF}}}$$

The standard flood‐field correction method has the effect of removing some beam profiles from the EPID images, such as “horns” induced by the flattening filter. A beam profile correction matrix was generated by using the field measurement data from water scan measurement data with open beam. Delivery with different total monitor unit and different dose rate were also tested in a previous study.^16^ The results exhibited good MU linearity and the dose rate dependency was found to be less than 1%.

### 2.B Conversion of the EPID raw images to incident photon fluence

To determine the incident photon beam fluence, it was necessary to simulate and calibrate the EPID device to establish a relationship between EPID pixel values and radiation dose. Detailed structure and composition of the EPID were provided by the manufacturer and were modeled using the GATE (Geant4 Application for Tomographic Emission), a GEANT4‐based Monte Carlo simulation platform.^17^ The source model of photon energy spectrum used in the MC simulations was based on the energy integration method and was evaluated using treatment planning systems (TPS) beam commissioning data. The scintillator layer of the EPID was made of phosphor terbium‐doped gadolinium oxysulphide (Gd_2_O_2_S: Tb) which was mostly used in EPIDs to convert the incident radiation beam to an optical signal. In order to accurately simulate the physics response of the EPID to photon beams, the optical photons tracking function was activated in the MC simulation process. The physical process of MV photon beam in the EPID dosimetry system is accurately simulated from the production of electrons in the build‐up layer. Then, the energy deposition in the GOS scintillator plate was recorded and the generation of optical photons initiated. Finally the optical simulation module simulates the transport of the optical photon in fibers and tallies the absorption of the optical photon in the amorphous silicon active TFT/diode array. The optical properties such as the surface type and refractive index were defined and stored in a table for simulation. Optical photons were detected by using a dielectric‐metal boundary and a digitizer was set‐up to record and analyze the optical absorption.

With the detail EPID modeled using GATE, a deconvolving kernel K_de_(x, y) was generated. The incident photon fluence Ψ*_p_*(x, y) on EPID can thereafter be reconstructed from the corrected EPID raw image and the K_de_(x, y) using the flowing equation

(2) $$\Psi_{p}\left(\text{x, y}\right)=\left({\bar{\text{EPID}}}_{\mathrm{raw}}{|}_{\mathrm{corrected}}\left(\text{x, y}\right)\right){⊗}^{-1}\left(K_{\mathrm{de}}\left(\text{x,y}\right)\right)$$

### 2.C Conversion of the reconstructed incident fluence to water‐based relative dose distribution

In practice, a water‐based dose distribution is measured using different detectors such as an ion chamber, diode, or film with plastic water phantoms for routine dosimetry measurements and patient‐specific QA because of the ease of set‐up and the reproducibility of chamber or film depth. As the flat panel of EPID was made from non‐water‐equivalent materials, in order to build a water‐based dose distribution from the incident photon fluence map reconstructed from the EPID measurement, a pencil‐beam dose kernel K_pb_(x, y) was simulated using the MCNPX code version 2.6.^18^ 2D dose distributions at d_max_ depths for all available photon energies were simulated in the MCNPX and specific number of source photons was selected to ensure an acceptable level of statistical uncertainty (< 1% at 3 cm off pencil beam, < 3% at 10 cm off axis for each simulation). The incident photon fluence map Ψ*_p_*(x, y) reconstructed from EPID raw measurement was then convolved with the K_pb_(x, y) to generate a two‐dimensional (2D) relative dose distribution in water at different d_max_ depths using

(3) $$D_{w}\left(\text{x, y}\right)=\Psi_{p}\left(\text{x, y}\right)⊗K_{\mathrm{pb}}\left(\text{x, y}\right)$$

The photon beams of the TrueBeam STx linac used in this study were calibrated to deliver 1 cGy/MU at the depth of dose maximum (d_max_) under reference setup condition of a 10 × 10 cm^2^ reference field with a nominal SSD of 100 cm. To determine the absolute doses, 100 MU (100 cGy) was delivered to the EPID with the same reference setup for cross calibrations. The EPID‐measured image data was recorded as D_EPID_ and an absolute calibration factor F_*ABS*_ for each energy was then calculated by calculating the ratio of 100 cGy and

(4) $$F_{ABS}=\frac{100\;\text{cGy}}{D_{\mathrm{EPID}}}$$

These calculated F_*ABS*_ were used to convert the EPID‐reconstructed relative dose into a water‐based absolute 2D dose.

All simulations in this study were run on a Linux server computer with 64 cores AMD Opteron central processing units (CPUs) and 128GB random‐access memory (RAM). A typical run generally took 2–4 h to yield statistically acceptable results without any effort on acceleration.

### 2.D System validation via standard fields

To validate the Monte Carlo simulation of EPID dosimetry system for photon beam application, series of tests with static radiation fields were performed for all five available energies (6 MV, 10 MV, 15 MV, and 6 MV FFF, 10 MV FFF). Standard square fields ranging from 4 × 4 to 15 × 15 cm^2^, MLCs formed circular fields of 2–15 cm in diameter, rectangular fields and irregular fields were tested. The EPID measured central axis absolute dose, and 2D off‐axis dose distributions at d_max_ in water were compared with water scan results using Farmer type ion chamber (PTW, Freiburg Germany) with vented sensitive volumes of 0.6 cm^3^, and measurements using and PTW729 ion chamber array. Farmer chamber and PTW 729 measurements were performed independently at a d_max_ depth of 1.5 cm for 6 MV and 6 MV FFF, 2.5 cm for 10 MV and 10 MV FFF, 3.0 cm for 15 MV. Off‐axis results were compared using γ‐index analysis. For all the γ‐index analysis in this study, measurement points of dose greater than 10% of the maximum planned dose are included and the dose difference criteria are based on global dose maximum.

### 2.E Clinical case study

A total of 12 patient plans with 6 MV, 10 MV, or 15 MV WFF beams were studied to evaluate the accuracy of the proposed EPID dosimetry method. Comparison of the results with PTW measurement and treatment planning calculations was carried out. In addition, 11 patient plans with high dose rate FFF beams of 6 MV or 10 MV for SBRT of lung, pancreas, liver, and pelvis (6–25 Gy per fraction, field sizes down to ~3 × 3 cm^2^) were also studied. 2D absolute dose maps were generated from EPID images using the proposed technique. The γ‐analysis was performed between EPID measurement, PTW729 measurement, and TPS calculation.

## 3 Results and discussion

### 3.A Monte Carlo simulation of pixel‐by‐pixel response of the EPID

**Figure 2:**
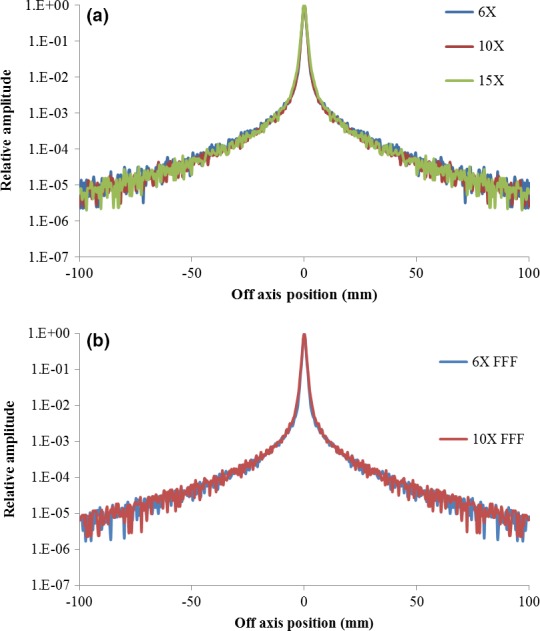
The dose‐glare kernel K_dp_(x, y) of all available WFF (a) and FFF (b) photon energies for deconvolution of EPID‐measured raw images into incident primary photon fluence.

A deconvolving dose kernel K_dp_(x, y), accounting for the MV photon dose deposition in EPID screen and the optical photon creation and scattering process, was generated from GATE MC simulations. The incident photon fluence on the EPID was then reconstructed from the corrected EPID raw images using Eq. (2). Figure 2 (a) and (b) show the change in K_dp_(x, y) as a function of distance from the central axis for WFF and FFF beams, respectively.

**Figure 3:**
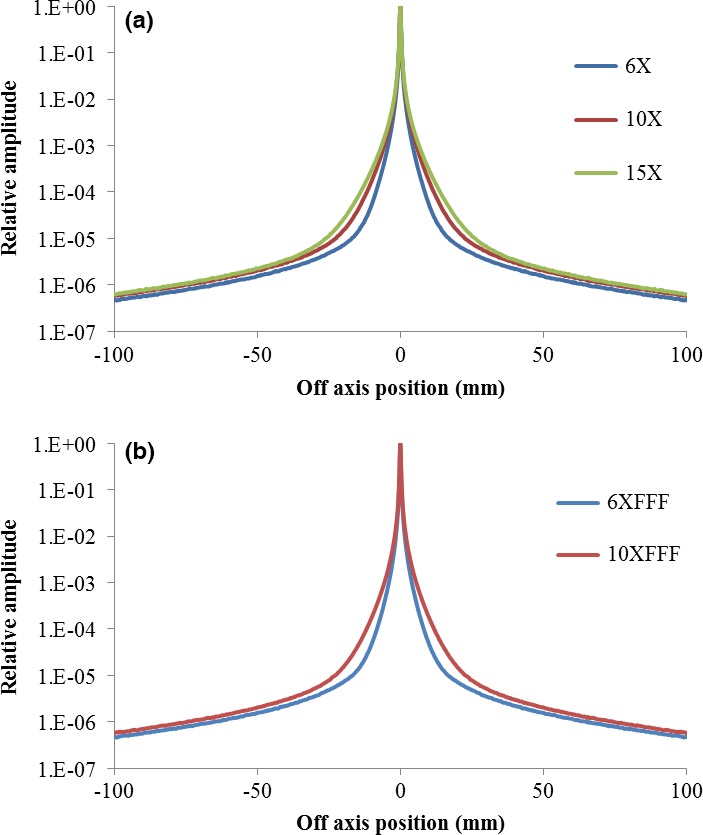
The water‐equivalent dose kernel K_pb_(x, y) of all available WFF (a) and FFF (b) photon energies for the reconstruction of water‐based dose distribution from the deconvoluted incident primary fluence.

The MCNPX simulated pencil‐beam dose kernels K_pb_(x, y) converted EPID images to 2D dose distribution in water as described in Sec 2C and the results are shown in (Fig. 3) for all available energies.

### 3.B System validation via standard fields

**Figure 4:**
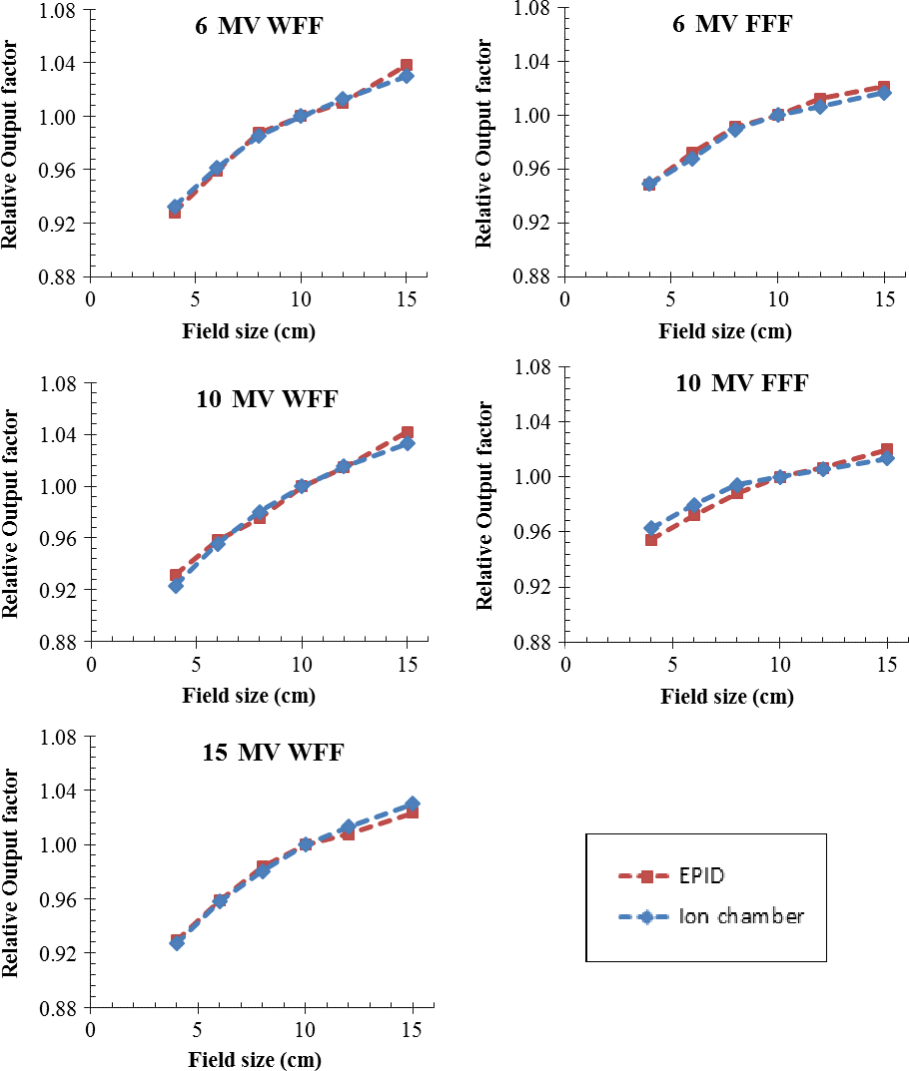
Output factors of 4 × 4, 6 × 6, 8 × 8, 10 × 10, 12 × 12, and 15 × 15 cm^2^ fields for all photon energies (6 MV, 10 MV, and 15 MV WFF, 6 MV and 10 MV FFF) measured with EPID and an ion chamber. The ion chamber measurement depth is 1.5 cm for 6 MV and 6 MV FFF, 2.5 cm for 10 MV and 10 MV FFF, 3.0 cm for 15 MV.

In Fig. 4, EPID‐measured output factors of different field sizes are shown along with that obtained using Farmer chamber. Overall, the output factors of square fields obtained using the two approaches agreed within 0.85%. The average discrepancy was found to be 0.02% ± 0.46% (mean ± standard deviation), 0.24% ± 0.53%, 0.10% ± 0.40%, −0.16% ± 0.56%, and 0.25% ± 0.59% for 6, 10, 15 MV WFF, and 6, 10 MV FFF photon beams, respectively. For all photon energies and for circular fields (2–15 cm diameter), rectangular and irregularly shaped fields, the EPID measured relative output factor or central axis dose output were found to agree with the ion chamber measurements to within 1.5%.

**Figure 5:**
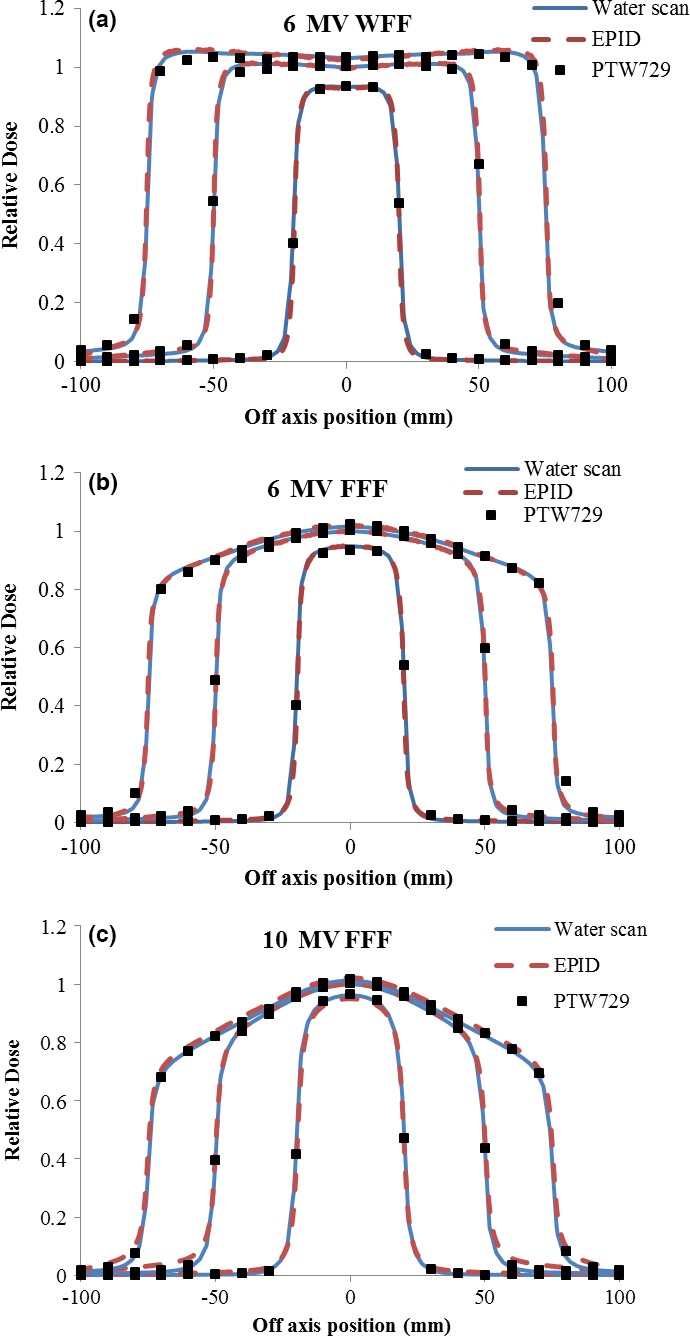
Dose profiles obtained using water scanning, ion chamber array, and EPID for (a) 6 MV WFF, (b) 6 MV FFF, and (c) 10 MV FFF photon beams of 4 × 4 cm^2^, 10 × 10 cm^2^, and 15 × 15 cm^2^.

In Fig. 5, we show the EPID‐measured dose profiles of various square 6 MV fields. The data obtained using a PTW729 detector and water tank scan are also plotted for comparison. Overall, the profiles obtained using different approaches agree each other very well. Small discrepancies (< 3%) were observed in the shoulder and trail regions of the profiles, presumably because of the PTW729 ion chamber array has more volume averaging effects and lateral scatter equilibrium problems due to the air cavities of the air filled ion chamber array. Therefore, the PTW729 result may not perfectly agree with the water scan result of a single pinpoint ion chamber and EPID‐converted dose profile.

To further validate the 2D accuracy of the EPID measurement against PTW729 ion chamber array measurement, γ‐index criteria of 3%/3 mm, 2%/2 mm, and 1%/1 mm were calculated for all WFF and FFF beams. The results, as presented in (Table 1), showed that greater than 99.4% passing rate for the criteria of 3%/3 mm for all energy modes. For the 2%/2 mm and 1%/2 mm γ criterion, greater than 92.3% and 67.9% passing rates were achieved, respectively. Similar γ‐index test results were found in measurements of circular fields of 2–15‐cm diameter, rectangular fields and irregular fields of all photon energies.

**Table 1:** γ‐index off‐axis analysis of EPID and PTW ion chamber array measurements

Field size [cm^2^]	6 MV WFF γ‐index pass rate (%)			6 MV FFF γ‐index pass rate (%)		
	3 mm/3%	2 mm/2%	1 mm/1%	3 mm/3%	2 mm/2%	1 mm/1%
4 × 4	100	96.3	75.4	99.5	92.5	67.9
10 × 10	100	99.8	87.2	99.7	97	82.2
15 × 15	100	99.9	92.3	100	98.8	90.2
	**10 MV WFF γ‐index pass rate (%)**			**10 MV FFF γ‐index pass rate (%)**		
4 × 4	99.9	96.1	74.3	99.6	92.3	68.2
10 × 10	100	99.2	86.6	99.8	97.4	81.7
15 × 15	99.8	99.4	91.5	100	98.4	90.1
	**15 MV WFF γ‐index pass rate (%)**					
4 × 4	99.6	96.6	73.9			
10 × 10	99.7	98.3	85.8			
15 × 15	99.4	98.1	91.2			

### 3.C Clinical case study

**Figure 6:**
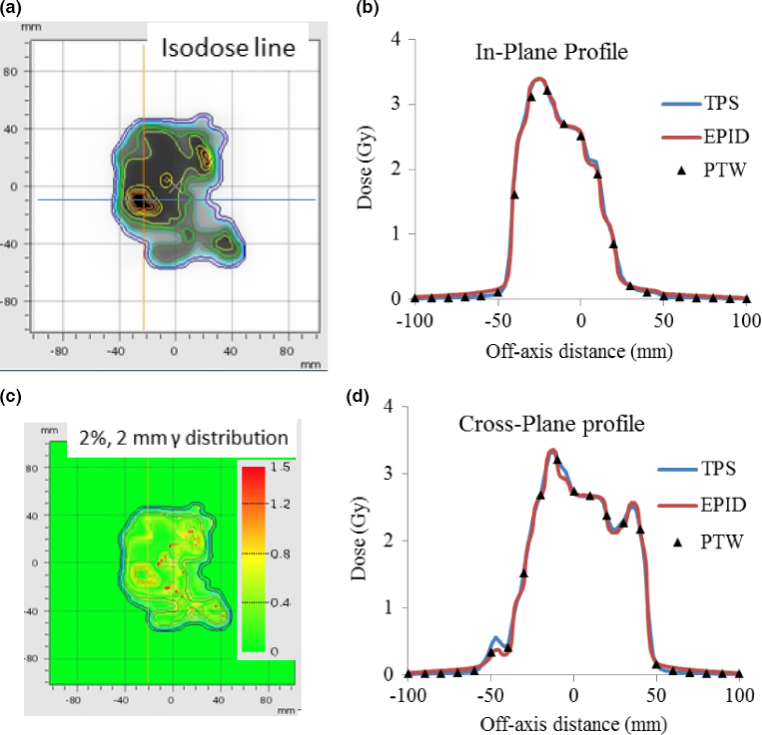
Patient case 1 6 MV WFF IMRT plan measured with EPID on treatment couch: (a) Isodose line overlay of EPID measurement and TPS calculation; (b) In‐plane profiles of EPID, PTW729 measurements, and TPS calculation; (c) 2 mm/2% γ‐index (between EPID measurements and TPS calculation) distribution map; and (d) Cross‐plane profiles of EPID, PTW729 measurements and TPS calculation.

**Figure 7:**
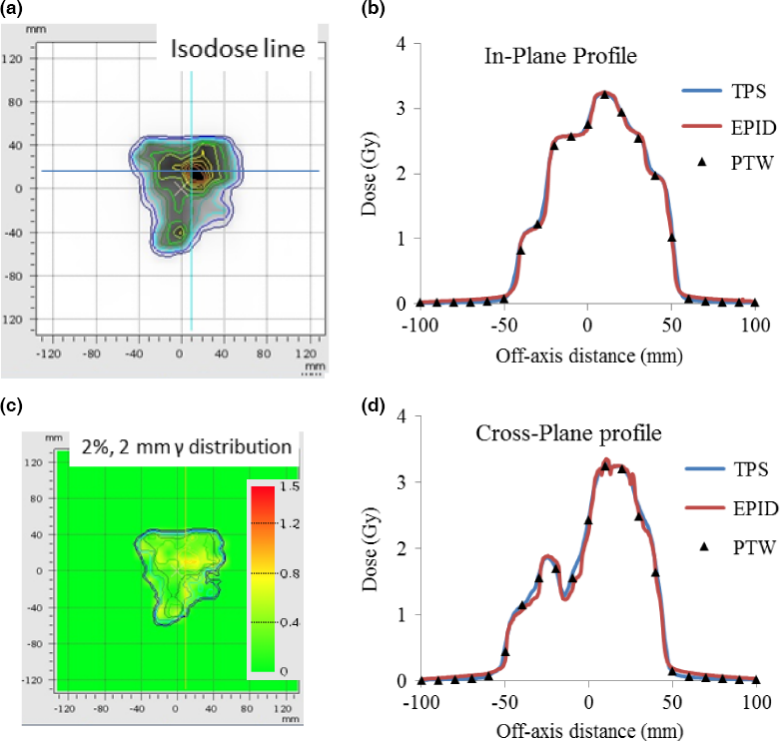
Patient case 2, 15 MV VMAT plan measured with EPID attached to the gantry: (a) Isodose line overlay of EPID measurement and TPS calculation; (b) In‐plane profiles of EPID, PTW729 measurements and TPS calculation; (c) 2 mm/2% γ‐index (between EPID measurements and TPS calculation) distribution map; and (d) Cross‐plane profiles of EPID, PTW729 measurements, and TPS calculation.

**Figure 8:**
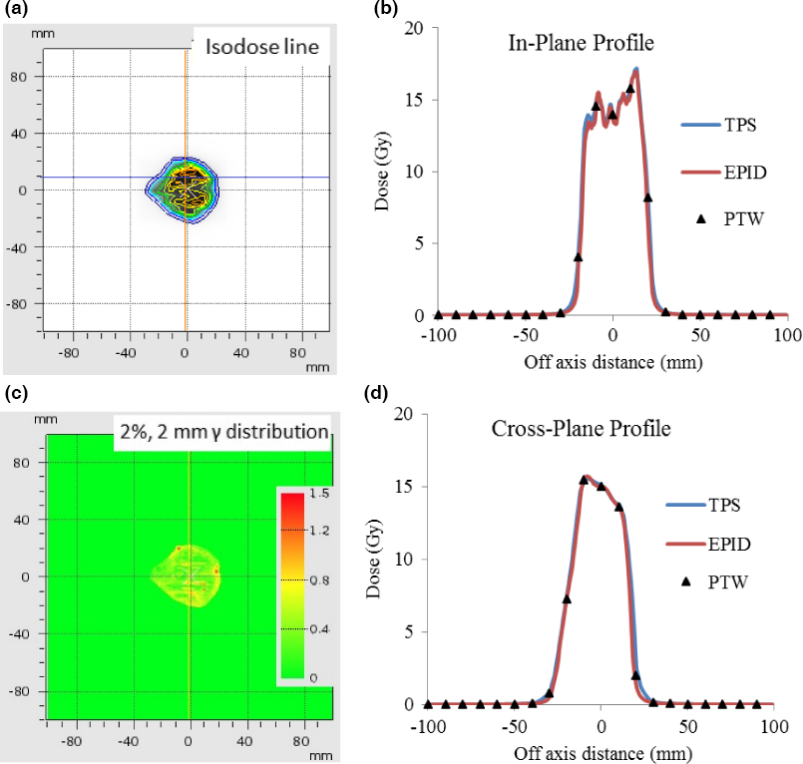
Patient case 3, 10 MV FFF rotational SBRT plan measured with EPID attached to the gantry: (a) Isodose line overlay of EPID measurement and TPS calculation; (b) In‐plane profiles of EPID, PTW729 measurements, and TPS calculation; (c) 2 mm/2% γ‐index (between EPID measurements and TPS calculation) distribution map; and (d) Cross‐plane profiles of EPID, PTW729 measurements, and TPS calculation.

Figures 6, 7, and 8 illustrate the EPID‐measured 2D isodose distributions, dose profile, and γ‐index analysis results for three typical clinical cases. The data obtained using a PTW729 detector array and TPS calculation are also presented. Overall, all three data sets showed good agreement for both cases. However, because of much higher density of detectors in the EPID (0.2 mm pixel size) as compared to that of the PTW729 (10 mm pixel size), more details of the dose distribution are revealed by the EPID, which is particularly valuable for small fields and/or for dosimetric measurement in high dose gradient region. Smallest grid size of 1 mm was set for all TPS dose calculations to ensure the 1%/1 mm gamma analysis. For the first case of a 6‐MV WFF IMRT plan measured with EPID on treatment couch, the γ‐index pass rates, between EPID measurement and TPS calculation, were 99.4%, 97.9%, and 78.0% for criterion of 3%/3 mm, 2%/2 mm, and 1%/1 mm, respectively. For the second case of a 15 MV VMAT plan measured with EPID attached to the gantry, the γ‐index pass rates were 100%, 100%, and 70.4% for criterion of 3%/3 mm, 2%/2 mm, and 1%/1 mm, respectively. The third case was a 10 MV FFF rotational SBRT plan measured with EPID attached to the gantry, the γ‐index pass rates were 100%, 98.7%, and 64.2% for criterion of 3%/3 mm, 2%/2 mm, and 1%/1 mm, respectively.

The γ‐index calculated based on the dosimetric difference between EPID and PTW measurements for a total of 23 patient cases shows an average passing rate of 99.2 ± 0.6%, 97.4 ± 2.4%, and 72.6 ± 8.4%, respectively for three prechosen γ‐index criterions: 3 mm/3%; 2 mm/2%; 1 mm/1%. For the γ‐index setting of 3 mm/3%, the minimum and maximum passing rates were 97.5% and 100%, respectively.

## 4 Conclusions

We have developed an EPID‐based dosimetric system based on the use of a Monte Carlo‐generated pixel response of the system. The EPID‐measured absolute dose distribution and output factors for standard square fields ranging from 4 × 4 to 15 × 15 cm^2^ were found to agree well with ion chamber data. The off‐axis measurement of the EPID was also found to be consistent with PTW729 and water scan data. For the clinical cases with various field sizes, the agreement between EPID‐ and PTW729‐measured values were found to be better than 2.1%. The success of EPID‐based system was also supported by the γ index analysis. The proposed EPID dosimetric system addresses an important unmet clinical need for an efficient and reliable dose measurement and verification in modern RT.

## Acknowledgment

This work is partially supported by NIH (1R01 CA133474 and 1R01 EB016777).
